# Risk factors for acute diarrhoea in children between 0 and 23 months of age in a peri-urban district of Pakistan: a matched case–control study

**DOI:** 10.1093/inthealth/ihac022

**Published:** 2022-05-14

**Authors:** Kamran Sadiq, Fatima Mir, Uswa Jiwani, Suhail Chanar, Apsara Nathwani, Muhammad Jawwad, Amjad Hussain, Arjumand Rizvi, Shah Muhammad, Muhammad Atif Habib, Sajid Bashir Soofi, Shabina Ariff, Zulfiqar A Bhutta

**Affiliations:** Department of Pediatrics and Child Health, Aga Khan University, Stadium Road, Karachi 74800, Pakistan; Department of Pediatrics and Child Health, Aga Khan University, Stadium Road, Karachi 74800, Pakistan; Center of Excellence in Women and Child Health, Aga Khan University, Karachi, Pakistan; Department of Pediatrics and Child Health, Aga Khan University, Stadium Road, Karachi 74800, Pakistan; Department of Pediatrics and Child Health, Aga Khan University, Stadium Road, Karachi 74800, Pakistan; Center of Excellence in Women and Child Health, Aga Khan University, Karachi, Pakistan; Center of Excellence in Women and Child Health, Aga Khan University, Karachi, Pakistan; Center of Excellence in Women and Child Health, Aga Khan University, Karachi, Pakistan; Center of Excellence in Women and Child Health, Aga Khan University, Karachi, Pakistan; Department of Pediatrics and Child Health, Aga Khan University, Stadium Road, Karachi 74800, Pakistan; Center of Excellence in Women and Child Health, Aga Khan University, Karachi, Pakistan; Department of Pediatrics and Child Health, Aga Khan University, Stadium Road, Karachi 74800, Pakistan; Center of Excellence in Women and Child Health, Aga Khan University, Karachi, Pakistan; Department of Pediatrics and Child Health, Aga Khan University, Stadium Road, Karachi 74800, Pakistan; Center of Excellence in Women and Child Health, Aga Khan University, Karachi, Pakistan

**Keywords:** diarrhoea, morbidity, mortality, risk factor, rural, Pakistan

## Abstract

**Background:**

Diarrhoea is a leading cause of preventable childhood morbidity and mortality worldwide. Unfortunately, Pakistan has the third-highest burden of diarrhoea-related deaths in children <5 y of age. Therefore we aimed to evaluate factors associated with diarrhoea among Pakistani children.

**Methods:**

A retrospective 1:2 matched case–control study nested in a baseline cross-sectional survey was conducted from October to December 2018 in Taluka Kotri, a two-thirds urban locality in the Jamshoro district. Children between the ages of 0 and 23 months with a history of diarrhoea in the 2 weeks preceding the survey were labelled as cases. Age-matched controls were children without symptoms of diarrhoea. Univariate and multivariable conditional logistic regression was performed to identify diarrhoea-related factors.

**Results:**

A total of 1558 cases were matched with 3116 controls. Factors significantly associated with lower odds of diarrhoea in the multivariate analysis included increasing maternal age (odds ratio [OR] 0.78 [95% confidence interval {CI} 0.67 to 0.90]), breastfeeding (OR 0.77 [95% CI 0.66 to 0.90]), higher paternal education (OR 0.79 [95% CI 0.65 to 0.97]) and belonging to the rich (OR 0.66 [95% CI 0.54 to 0.80]) and richest quintiles (OR 0.54 [95% CI 0.44 to 0.66]).

**Conclusions:**

This study identifies risk factors associated with diarrhoea in children <23 months of age, including younger maternal age, higher paternal education, not breastfeeding and poverty, which has implications for developing preventive programs and strategies that target populations with a higher risk of diarrhoea.

## Introduction

Globally, there are nearly 1.7 billion cases of childhood diarrhoeal disease, causing 525 000 deaths each year.^1^ Children from the developing world are disproportionately affected by diarrhoea and experience, on average, three diarrhoeal episodes every year.^1,2^ Repeated episodes of diarrhoea cause both acute morbidity and long-term adverse health outcomes, including malnutrition, linear growth stunting and impaired neurodevelopment.^3,4^ Across low- and middle-income countries (LMICs), the incidence of diarrhoea in children <5 y of age is highest in sub-Saharan Africa, with 371 million episodes per year, and South Asia, with 228 million episodes per year.^5^ The majority of diarrhoea-related mortality is similarly clustered in these two regions, with an estimated 101 927 and 290 724 deaths occurring in South Asia and sub-Saharan Africa, respectively.^5^ Although there is a high burden of childhood mortality due to diarrhoea, most of these deaths can be avoided by addressing inequities in intervention coverage.^6^

The 2017–2018 Pakistan Demographic and Health Survey (PDHS) reported the national prevalence of diarrhoea among children <5 y of age to be 19%.^7^ Pakistan has the third-highest burden of diarrhoea-related mortality globally, with 39 500 deaths in children <5 y of age attributed to diarrhoea annually.^8^ In addition, the World Health Organization’s integrated Global Action Plan for Pneumonia and Diarrhoea (GAPPD) identified the country as one of 15 high-burden focus countries.^9^ Tackling pneumonia and diarrhoea in Pakistan and other high-burden countries is crucial for ending preventable childhood deaths due to pneumonia and diarrhoea.^9^

Previous studies have identified inadequate water, sanitation and hygiene (WASH) practices,^10^ not breastfeeding,^11^ age <24 months^12,13^ and low maternal education^14^ as risk factors for childhood diarrhoea. Interventions that promote immunization, handwashing with soap, safe sanitation, water purification and safe water storage practices have previously been shown to effectively reduce diarrhoeal diseases in children.^15–19^ However, risk factors for diarrhoea vary across and within countries, showing that there is no universal intervention that can reduce childhood diarrhea.^20,21^ Moreover, assessment of risk factors of diarrhoeal mortality at the national level can hide subnational variations, providing limited evidence for policymaking.^22^ Therefore, estimating diarrhoea risk factors at a spatial scale can enable the planning and implementation of context-specific strategies to reduce diarrhoea. To better understand the factors associated with childhood diarrhoea in rural Pakistan, we surveyed the Jamshoro district, located in the province of Sindh. We included children ages 0–23 months because they have the highest mortality risk.^13^

## Methods

### Study design and setting

We conducted a retrospective 1:2 case–control study nested within a baseline cross-sectional survey conducted before a cluster randomized controlled trial (protocol under publication). Jamshoro has a literacy rate of 33% and more than half of the people are daily wage laborers. There are 27 health facilities in the district, including one district headquarter (DHQ) hospital, three taluka headquarter (THQ) hospitals, five rural health centres (RHCs) and 18 primary health units (BHUs). Taluka Kotri is two-thirds urban and has an estimated population of 0.44 million people (44% of the district's population), with approximately 64 500 children <5 y of age.

### Study population

We included children between 0 and 23 months of age within 10 union councils (UCs) of Taluka Kotri. Participants were included if they were registered with the Lady Health Worker (LHW) Program by the Department of Health in Sindh. LHWs are a community-based cadre of healthcare workers who provide essential primary health services in rural and urban slums. Participants were excluded if information regarding their breastfeeding or immunization status was missing. A case was defined as a child between 0 and 23 months of age who had an episode of diarrhoea 2 weeks before the survey as reported by the mother or primary caregiver. Diarrhoea was defined according to the standard definition used in the Multiple Indicator Cluster Surveys as three or more loose stools in 24 h or more frequently than is normal for the child.^23^ Two controls were matched with each case based on the child's age in months. Definitions used for sociodemographic and WASH variables are presented in Table 1.

**Table 1.** Definitions for sociodemographic and WASH variables

Sociodemographic variables	Overcrowding	Defined as six or more people living in one room
	Wealth index	A composite score was created as a measure of socio-economic status based on a range of consumer goods owned by households (television, refrigerator, mobile phone, internet, bed, etc.), housing characteristics (source of drinking water, type of sanitation facilities, flooring material, access to electricity, number of rooms used for sleeping, fuel used for cooking) and ownership of assets, land, and livestock. According to these indicators, wealth scores were generated and surveyed households were ranked according to their wealth score
Behavioural and WASH practices	Improved source of drinking water	Includes piped water, public taps, tube wells, boreholes, protected dug wells, springs, rainwater and bottled water
	Improved source of water for usage	Includes piped water, public taps, tube wells, boreholes, protected dug wells, springs and rainwater
	Improved source of sanitation	Includes non-shared toilets that are flush/pour toilets to piped sewer systems, septic tanks and pit latrines
	Appropriate hand washing practices	Includes washing hands before and after eating, before feeding baby, before cooking, after fieldwork, after using the toilet and after changing diapers

### Data collection

Data were collected from October 2018 to December 2018 by independent community health workers (CHWs) who accompanied LHWs on their routine household visits. There were 29 258 households registered with the LHW Program in the 10 UCs, covered by 210 LHWs. In addition, 6657 homes had at least one child between 0 and 23 months of age. Convenience sampling was adopted for data collection of the baseline survey, where households that were accessible to CHWs by foot or vehicle and had a respondent available at the time of the study were included in the study. CHWs used a structured, paper-based questionnaire to interview mothers/primary caregivers in each household in the survey area to identify cases and controls. If there was more than one case in the home, the child with the most recent episode of diarrhoea was included. The need for inpatient treatment was used to assess the severity of diarrhoea. If the LHW who accompanied the CHW identified a sick child, she evaluated the severity of the disease. In addition, she provided immediate care, instructions for home-based management and referral if needed.

The children with a history of diarrhoea identified in the baseline survey were included as cases in the study. All children who did not have diarrhoea were matched with cases based on age in completed months (difference: 1 month) using Stata version 16 (StataCorp, College Station, TX, USA). Identified age-matched controls were subsequently randomly selected. Children were considered to be fully immunized if they had received all the vaccines specified by the government of Pakistan's Expanded Program on Immunization (EPI) schedule.^24^ Data for each vaccine in the EPI schedule were collected separately. The data were rechecked and double entered to ensure accuracy.

### Statistical analysis

Between October and December 2018, a total of 1558 cases and 3116 matched controls were recruited to the study. This gives a 90% power for detecting an odds ratio (OR) ≥1.5 as significant at the 5% level if the prevalence of exposure among controls is 10–90% and correlation between cases and controls is 0.2–1.^25^ All data analyses were conducted using Stata version 16. We performed descriptive analyses presented as frequencies and proportions. Factors associated with diarrhoea were determined using univariate conditional logistic regression. Variables with p<0.2 were then included in a multivariable regression model and fit backward elimination. Results are presented as adjusted matched ORs (amORs) with 95% confidence intervals (CIs). p-Values <0.05 were considered statistically significant.

### Ethics statement

All caretakers of the children included in the study provided informed and signed consent. If the caretaker was illiterate, we obtained signed permission through a thumb impression in the presence of a witness. In the case where a household was locked and could not be approached, or if the household members refused to consent, the following household with an eligible child was selected. The Ethics Review Committee at the Aga Khan University approved the study (4722-Ped-ERC-17).

## Results

A total of 4674 children between the ages of 0 and 23 months were included in this study. A total of 1558 cases were matched with 3116 controls. The demographic characteristics are presented in Table 2. In unadjusted analysis, children of mothers >30 y of age had lower diarrhoea odds than children of mothers <25 y of age (OR 0.74 [95% CI 0.63 to 0.86]). Children also had lower odds of diarrhoea if their mothers (OR 0.66 [95% CI 0.54 to 0.80]) and fathers attained higher education (OR 0.63 [95% CI 0.53 to 0.74]). In addition, children belonging to the rich (OR 0.69 [95% CI 0.57 to 0.83]) and wealthiest quintile (OR 0.54 [95% CI 0.45 to 0.66]) were less likely to have diarrhoea than those from the poorest quintile.

**Table 2.** Univariate analysis of demographic characteristics and their associations with diarrhoea

Variable	Cases, n (%)	Controls, n (%)	OR (95% CI)	p-Value
Gender				
Male	790 (50.7)	1588 (51.0)	Ref	Ref
Female	768 (49.3)	1528 (49.0)	1.01 (0.89 to 1.14)	0.868
Household density				
0-6	1292 (82.9)	2597(83.3)	Ref	Ref
> = 7	266 (17.1)	519 (16.7)	1.03 (0.88 to 1.21)	0.719
Mother's age				
≤25	555 (35.7)	943 (30.4)	Ref	Ref
25-30	540 (34.8)	1121 (36.1)	0.81 (0.70 to 0.94)	0.007
>30	459 (29.5)	1043 (33.6)	0.74 (0.63 to 0.86)	<0.001
Mother's education				
No education	877 (56.4)	1584 (51.0)	Ref	Ref
Primary	232 (14.9)	459 (14.8)	0.91 (0.76 to 1.09)	0.291
Secondary	272 (17.5)	586 (18.9)	0.85 (0.72 to 0.99)	0.049
Higher	173 (11.1)	478 (15.4)	0.66 (0.54 to 0.80)	<0.001
Father's education				
No education	536 (34.8)	891 (28.9)	Ref	Ref
Primary	254 (16.5)	473 (15.3)	0.89 (0.74 to 1.08)	0.247
Secondary	440 (28.5)	892 (28.9)	0.83 (0.71 to 0.97)	0.017
Higher	312 (20.2)	829 (26.9)	0.63 (0.53 to 0.74)	<0.001
Wealth index (quintiles)				
Poorest	361 (23.2)	574 (18.4%)	Ref	Ref
Poor	341 (21.9)	594 (19.1%)	0.90 (0.75 to 1.09)	0.293
Middle	335 (21.5)	600 (19.3%)	0.87 (0.73 to 1.05)	0.163
Rich	283 (18.2)	652 (20.9%)	0.69 (0.57 to 0.83)	<0.001
Richest	238 (15.3)	696 (22.3%)	0.54 (0.45 to 0.66)	<0.001

Ref: reference.

Table 3 shows the breastfeeding and WASH practices and their associations with diarrhoea. Feeding colostrum (OR 0.80 [95% CI 0.65 to 0.97]) and any breastfeeding at the time of the survey (OR 0.79 [95% CI 0.68 to 0.92]) were associated with lower odds of diarrhoea. In addition, children living in households with access to an improved source of drinking water (OR 0.84 [95% CI 0.72 to 0.97]) and improved source of water for usage (OR 0.84 [95% CI 0.73 to 0.98]) were less likely to have diarrhoea compared with children living in houses with unimproved sources.

**Table 3.** Univariate analysis of breastfeeding and WASH practices and their associations with diarrhoea

Variable	Cases, n (%)	Controls, n (%)	OR (95% CI)	p-Value
Colostrum given to baby				
No	173 (11.1)	283 (9.1)	Ref	Ref
Yes	1385 (88.9)	2833 (90.9)	0.80 (0.65 to 0.97)	0.027
Currently breastfeeding				
No	387 (24.8)	656 (21.0)	Ref	Ref
Yes	1171 (75.2)	2460 (78.9)	0.79 (0.65 to 0.97)	0.002
Exclusively breastfed				
No	1218 (78.2)	2373 (76.2)	Ref	Ref
Yes	340 (21.8)	743 (23.8)	0.89 (0.77 to 1.03)	0.122
Cooking area				
Indoor	1503 (96.5)	3040 (97.6)	Ref	Ref
Outdoor	55 (3.5)	76 (2.4)	1.46 (1.03 to 2.07)	0.035
Improved source of drinking water				
No	365 (23.4)	635 (20.4)	Ref	Ref
Yes	1193 (76.6)	2481 (79.6)	0.84 (0.72 to 0.97)	0.017
Improved source of water for usage				
No	377 (24.2)	662 (21.3)	Ref	Ref
Yes	1181 (75.8)	2454 (78.8)	0.84 (0.73 to 0.98)	0.022
Any measures taken to make drinking water safe				
No	720 (46.2)	1420 (45.6%)	Ref	Ref
Yes	838 (53.8)	1696 (54.4%)	0.97 (0.86 to 1.10)	0.676
Appropriate hand washing practices				
No	1455 (93.4)	2898 (93.0%)	Ref	Ref
Yes	103 (6.6)	218 (7.0%)	0.94 (0.74 to 1.20)	0627
Improved source of sanitation				
No	1243 (79.8)	2536 (81.4%)	Ref	Ref
Yes	315 (20.2)	580 (18.6%)	1.11 (0.95 to 1.29)	0.190

Ref: reference.

According to the EPI schedule, only 34 (2.2%) children with diarrhoea and 75 (2.4%) children without diarrhoea were fully vaccinated. This was due to the low uptake of rotavirus vaccine in the study area (cases: 11 [0.7%], controls: 15 [0.5%]; p=0.347). According to the EPI schedule, children who had completed their vaccinations had similar odds of contracting diarrhoea as children who were not fully immunized (OR 0.83 [95% CI 0.48 to 1.45]) (Table 4).

**Table 4.** Univariate analysis of immunization status and its association with diarrhoea

Variable	Cases, n (%)	Controls, n (%)	OR (95% CI)	p-Value
EPI vaccinations including rotavirus				
Incomplete	1524 (97.8)	3041 (97.6)	Ref	Ref
Complete	34 (2.2)	75 (2.4)	0.83 (0.48 to 1.45)	0.516
EPI vaccinations excluding rotavirus				
Incomplete	684 (43.9)	1291 (41.4)	Ref	Ref
Complete	874 (56.1)	1825 (58.6)	0.89 (0.78 to 1.02)	0.089

Ref: reference.

Multivariate analysis showed that children of mothers >30 y of age had lower diarrhoea odds than children of mothers <25 y of age (OR 0.73 [95% CI 0.63 to 0.86]) (Table 5). Breastfeeding (OR 0.76 [95% CI 0.65 to 0.89]) and higher paternal education (OR 0.79 [95% CI 0.65 to 0.97]) were protective against diarrhoea. Children from the rich (OR 0.73 [95% CI 0.59 to 0.91]) and richest quintile (OR 0.59 [95% CI 0.47 to 0.74]) had lower odds of diarrhoea than children from the poorest quintile.

**Table 5.** Multivariate analysis (adjusted and reduced) for factors associated with diarrhoea

	Adjusted		Reduced	
Variables	amOR (95% CI)	p-Value	amOR (95% CI)	p-Value
Mother's age				
≤25	Ref	Ref	Ref	Ref
25–30	0.82 (0.71 to 0.96)	0.013	0.82 (0.71 to 0.96)	0.013
>30	0.78 (0.66 to 0.91)	<0.001	0.73 (0.63 to 0.87)	<0.001
Mother's education				
No education	Ref	Ref	–	–
Primary	1.03 (0.85 to 1.24)	0.748	–	–
Secondary	1.03 (0.85 to 0.12)	0.782	–	–
Higher	0.97 (0.76 to 1.23)	0.792	–	–
Father's education				
No education	Ref	Ref	–	–
Primary	0.94 (0.77 to 1.14)	0.537	–	–
Secondary	0.93 (0.78 to 1.10)	0.406	–	–
Higher	0.80 (0.64 to 1.99)	0.038	–	–
Colostrum given to baby				
No	Ref	Ref	–	–
Yes	0.84 (0.69 to 1.10)	0.113	–	–
Currently breastfeeding			–	–
No	Ref	Ref	Ref	Ref
Yes	0.77 (0.66 to 0.90)	0.001	0.76 (0.65 to 0.89)	<0.001
Exclusively breastfed				
No	Ref	Ref	–	–
Yes	0.92 (0.79 to 1.07)	0.278	–	–
Cooking area				
Indoor	Ref	Ref	–	–
Outdoor	1.21 (0.84 to 1.)	0.314	–	–
Improved source of drinking water				
No	Ref	Ref	–	–
Yes	1.02 (0.73 to 1.42)	0.913	–	–
Improved source of water for usage				
No	Ref	Ref	–	–
Yes	0.96 (0.69 to 1.33)	0.794	–	–
Improved source of sanitation				
No	Ref	Ref	–	–
Yes	1.14 (0.98 to 1.34)	0.097	–	–
EPI vaccinations including rotavirus				
Incomplete	Ref	Ref	–	–
Complete	0.83 (0.48 to 1.45)	0.516	–	–
Wealth index (quintiles)				
Poorest	Ref	Ref	Ref	Ref
Poor	0.92 (0.76 to 1.11)	0.383	0.91 (0.75 to 1.10)	0.340
Middle	0.91 (0.74 to 1.11)	0.343	0.89 (0.73 to 1.09)	0.272
Rich	0.75 (0.60 to 0.94)	0.012	0.73 (0.59 to 0.90)	0.004
Richest	0.61 (0.47 to 0.79)	<0.001	0.59 (0.47 to 0.75)	<0.001

## Discussion

This study evaluated the factors associated with diarrhoea in children ages 0–23 months in a rural district of Pakistan. We found that breastfeeding was associated with a lower likelihood of diarrhoea in children. Breastfeeding is considered one of the most cost-effective interventions to reduce childhood morbidity and mortality.^26^ Evidence indicates that approximately half of all diarrhoea episodes and 72% of hospital admissions due to diarrhoea can be prevented by breastfeeding.^27^ A meta-analysis evaluating the impact of breastfeeding on diarrhoea-related morbidity and mortality showed that breastfed children ages 6–23 months had less than half the risk of dying due to diarrhoea than children who weren't breastfed.^28^ Another study from rural China found that breastfeeding reduced the risk of diarrhoea in children <2 y of age despite poor dietary diversity.^29^ In addition, breastmilk likely protects infants from infectious diseases.^30^

Similar to studies from other LMICs, including India^26^ and Bangladesh,^3^^1^ our study showed that a lower wealth index was associated with higher odds of diarrhoea. This finding may be because of the impact of socio-economic inequalities on health-seeking behaviour. People from poorer households are less likely to seek and utilize healthcare services,^3^^2^ including preventive services such as immunization.^3^^3^ Moreover, food insecurity and malnutrition, associated with diarrhea,^3^^4^,^3^^5^ are highly correlated with poverty.^3^^6^,^3^^7^

Our study reported that a mother's age was also correlated with childhood diarrhoea. Similar findings have been reported in studies from Ethiopia,^3^^8^ Tanzania^39^ and Burundi.^4^^0^ Poor health outcomes in children of younger mothers may be due to the social disadvantages more youthful mothers face.^4^^1^ Women from LMICs who have children at an early age are likely to remain poor and uneducated.^4^^2^ Moreover, 55 LMICs reported that first-born children of older women have better health outcomes regardless of socio-economic status.^4^^1^

We found that access to improved drinking water sources and water usage was not associated with diarrhoea. It may be because the current definition of improved water sources does not always predict that the water is safe from disease-causing microbes.^4^^3^ Factors such as unsafe storage, interrupted supply of piped water, an untreated source used for piped water supply and irregular use of the improved sources are all associated with microbial contamination and a subsequent increase in diarrhoea incidence.^4^^3^,^4^^4^ Similarly, our study did not show an association between improved sanitation and diarrhoeal disease. However, a randomized controlled trial in rural India demonstrated that less than universal coverage of improved sanitation practices in a community does not reduce the incidence of childhood diarrhoea.^4^^5^ In our study area, only 20% of the population had access to an improved source of sanitation. Moreover, improvements in sanitation alone do not interrupt transmission pathways of oral–faecal exposure, which is vital to prevent diarrhoeal diseases.^4^^6^

Our results demonstrated an alarmingly low uptake of the rotavirus vaccine in the study area, even though the coverage for all other vaccines was comparable with the national average.^7^ We did not find an association between diarrhoea and immunization. Previous studies have shown that rotavirus vaccination effectively prevents severe diarrhoea,^15^,^38^ hospitalization and mortality in young children.^4^^7^ Our results may be explained by the lower efficacy of oral vaccines in LMICs^4^^8^ and because we did not limit cases to severe diarrhoea.

Although the incidence of diarrhoea in Pakistan decreased by 12.7% between 2000 and 2016,^49^ the disease remains one of the leading causes of childhood morbidity and mortality in the country.^7^ Our study identified several factors that can be targeted to decrease the burden of diarrhoea. Interventions that promote immunization and breastfeeding are vital in reducing the incidence of diarrhoea and diarrhoea-related morbidity and mortality among children. However, it is also imperative to study how these interventions impact people from different socio-economic backgrounds since evidence suggests that merely increasing the coverage of an intervention may not bridge the gap in health outcomes across wealth quintiles.^50^

This study has several limitations. First, as this was an observational study, it was impossible to establish a causal relationship between diarrhoea and the identified risk factors. Another limitation is that the incidence of diarrhoea was determined by asking caregivers if their children had symptoms of diarrhoea 2 weeks before the survey, which may have introduced recall bias. Moreover, convenience sampling was utilized for the baseline survey. Finally, we considered only the most recent episode of diarrhoea for this study. However, the risk factors we evaluated are chronic exposures in this population and are likely associated with other diarrhoeal episodes. Additionally, our results may not be generalizable to completely rural or urban regions since the participants were from a regional area with both urban and rural populations.

## Conclusions

Pakistan has one of the highest burdens of diarrhoea and diarrhoea-related mortality globally. Our study identified that breastfeeding, older maternal age, higher paternal education and household wealth indicators are associated with lower odds of diarrhoea in children between the ages of 0 and 23 months. Therefore, promoting breastfeeding can potentially reduce diarrhoea among young children. Moreover, studying the effect of poverty on health outcomes and ensuring that interventions to reduce diarrhoea reach populations from all socio-economic strata are vital to reducing Pakistan's morbidity and mortality in children <5 y of age.

## Data Availability

All the data are available from the corresponding author upon reasonable request.
